# Oral Infection and Its Treatment in Army Camps

**Published:** 1919

**Authors:** 


***Oral Infection and its Treatment in Army Camps.*** By Lieut. HARRY LEE, D. C. *Journal Association Military Dental Surgeons of U. S.*, October 1918.

1. *Chronic Infection of the Mouth.*

In multi-rooted teeth where the peridental membrane is destroyed by a chronic abscess, if the diagnosis is confirmed by X-ray examination, the treatment is extraction and curettement of the pocket. The same treatment is given single-rooted teeth if the destruction of peridental membrane is extensive; where there is only a moderate amount of destruction, treatment is according to the conditions present.

2. *Chronic Ulcerative Stomatitis or Trench Mouth.*

Two bacteria are involved in etiology of this condition : fusiform bacillus and the spirochete of Vincent. The chief symptoms are ulcers on the gum margins of the teeth, base of ulcers overlaid with a grayish white covering, lymphatics related to the area enlarged and tender. Treatment : Clean the ulcer thoroughly, dry with aseptic cotton and apply $H_2O_2$ full strength; also have patient use $H_2O_2$ as a mouth wash three or four times daily. Hydrogen peroxide is used because the bacteria are anerobic. The following preparation may also be used as a mouth wash and spray:

| | |
|---|---|
| Hydrogen Peroxide . . . . . . . . . . . . | 5 oz. |
| Glycerine. . . . . . . . . . . . . . . . . . . . | 5 drachms. |
| Wine of Ipecac . . . . . . . . . . . . . . . . | 3 drachms. |
| Aqua dist. q. s. . . . . . . . . . . . . . . . | 8 oz. |

The author also recommends giving 1/4 grain calomel three times a day for a period of seven to ten days, unless symptoms of accumulation appear, and epsom salts four or five times weekly. Healing is much more slow without this internal treatment. In foreign camps Fowler's solution U. S. P. in doses of 2 to 5 minims is given internally. The author is going to try this with the $H_2O_2$ locally, and believes it will be very efficient.

